# Asymptomatic malaria infections and associated risk factors in malaria-eliminating settings of Nong District, Savannakhet Province, Lao People’s Democratic Republic

**DOI:** 10.1186/s41182-025-00702-y

**Published:** 2025-02-19

**Authors:** Taofic Bouwe, Daisuke Nonaka, Philippe Buchy, Parita Hansana, Boualam Khamlome, Vixayyang Chayvangmanh, Noudéhouénou Credo Adelphe Ahissou, Keobouphaphone Chindavongsa, Tiengkham Pongvongsa, Virasack Banouvong, Moritoshi Iwagami

**Affiliations:** 1 Department of Global Health, Graduate School of Health Sciences, University of the Ryukyus, Okinawa, 903-0215 Japan; 2 Institut Pasteur du Laos, Ministry of Health, Sansenthai Road, Ban Kao-Gnot, Sisattanak District, P.O. Box 3560, Vientiane Capital, Lao PDR; 3 Center of Malariology, Parasitology and Entomology (CMPE), Koualuangtai Village, Chanthabouly District, Capital of Vientiane, Lao PDR; 4 Division of Non-Communicable Disease Control, Department of Healthcare and Rehabilitation, Ministry of Health, Sisattanak District, Vientiane Capital, Lao PDR; 5 Savannakhet Provincial Health Office, Khanthabouly Road, Kaysone Phomvihanh City, Savannakhet Province Lao PDR; 6 Department of Tropical Medicine and Malaria, Research Institute, National Center for Global Health, and Medicine (NCGM), Tokyo, 162-8655 Japan

**Keywords:** Malaria, Asymptomatic, Prevalence, Loop-mediated isothermal amplification, Lao PDR, Elimination

## Abstract

**Background:**

As the Lao People’s Democratic Republic is nearing malaria elimination, asymptomatic malaria infections remain a challenge to address. Control measures focusing on symptomatic persons do not effectively work for asymptomatic infections which often go undetected by conventional diagnostic tools. It is therefore crucial to understand the burden of asymptomatic malaria for tailored interventions to eliminate the disease. This study assessed the prevalence of asymptomatic malaria infections with associated risk factors in an endemic district of Savannakhet province.

**Methods:**

In March 2024, a cross-sectional study was conducted in three villages of Nong District. Blood samples were collected from the fingertips of the participants for *Plasmodium* parasite identification using microscopy and Loop-mediated Isothermal Amplification (LAMP); those aged 13 years and above were also interviewed. Mann–Whitney *U* test and Fisher’s exact test were performed to compare the medians of different age and temperature groups and determine the association between predictor variables and outcome variables respectively.

**Results:**

A total of 622 individuals participated in this survey; *Plasmodium* parasites were detected in 2.1% (13/622) of participants. The prevalence of asymptomatic malaria was 1.8% (11/622). *Plasmodium vivax* accounted for 15.4% (2/13) of all positive cases. The remaining species could not be identified*.* Farmers aged 15 years and above accounted for 81.8% of the asymptomatic infections. Ninety percent (90%) of the participants used bed nets in the village. Among interviewed participants, 23.6% reported not using mosquito bed nets in the forest; 21.3% of the participants who had been to the forest were nighttime forest workers.

**Conclusions:**

This study revealed a prevalence of 1.8% of asymptomatic malaria infections in the study areas with the majority of the infections clustered among farmers, and an important proportion of these populations not using protective measures in the forest. These findings showed that malaria reservoirs are notable with a lack of use of protective measures, which could threaten malaria control and elimination efforts. Therefore, malaria elimination in Lao PDR by 2030 would need interventions targeting high-risk adult populations for screening with sensitive tools coupled with sensitization on protective measures and asymptomatic malaria.

**Supplementary Information:**

The online version contains supplementary material available at 10.1186/s41182-025-00702-y.

## Background

Malaria is a parasitic disease that remains an important public health threat. In 2022, around 249 million cases of malaria were recorded in malaria-endemic countries [1]. The Lao People’s Democratic Republic (Lao PDR) is one of the malaria-endemic countries in the Greater Mekong Subregion in Southeast Asia. With an estimated population of 7.3 million in 2021 [2], malaria incidence per 1,000 population at risk in Lao PDR in the same year was 1.7, a little higher than in 2020 (1.5) [3].

While malaria cases are recorded and reported by healthcare facilities throughout the year, there are increasing cases that reach high peaks during the rainy season. Although *Plasmodium falciparum* had long been predominant, the proportion of *Plasmodium vivax* infections has been rising recently [4]. Several risk factors of malaria infection have been reported from Lao PDR: groups that are most at risk are those living in farm huts at night without insecticide-treated nets (INTs), ethnic minorities, remote, hilly, and forest fringe inhabitants, and at night forest workers [5–8]. Soldiers also are at risk of malaria due to their training or patrols and camps in forests, especially along the border of the country [9]. Malaria protective measures in the country mostly include the use of insecticide-treated bed nets, mosquito repellent, and long-sleeved clothes [10]. During the past decade, Lao PDR has achieved sizeable progress in malaria control by reducing malaria cases by 80% (from 2016 to 2020), and no death case related to malaria has been reported since 2018 [11].

Despite this remarkable progress, there are challenges hindering malaria elimination by 2030 as set by the government of Lao PDR and the World Health Organization (WHO). One is the asymptomatic *Plasmodium* infections in southern provinces including Savannakhet province. A study conducted in this province in 2015, reported 20% (175/888) of asymptomatic malaria infections [12]. These infections are missed by microscopy and rapid diagnostic tests (RDTs) because of low parasite density [9] (< 50 parasites/µl) [12, 13].

Another challenge is passive case detection, whereby symptomatic persons are captured at healthcare facilities [14]. This approach does not work for capturing asymptomatic individuals who are unlikely to visit a healthcare facility due to the absence of symptoms. As such, asymptomatic individuals constitute malaria reservoirs and are a cause of malaria infections spread, because *Anopheles* mosquitoes feeding on blood from them can become infected and perpetuate malaria transmission [10]. Therefore, in countries close to malaria elimination such as Lao PDR, alternative approaches are crucial to interrupt the transmission. One such approach is active case detection using highly sensitive diagnostic tools to find clusters of asymptomatic persons and treat them [9, 15].

Active case detection, according to the definition by the WHO, is the detection by health workers of malaria infections at the community and household level in population groups that are considered to be at high risk [16]. Active case detection can include two different approaches: the first one, reactive case detection (RACD), is the process of finding other cases following an index case detected passively. The second one, proactive case detection (PACD), consists of screening the community at risk without a trigger of any malaria case. The strategy of PACD which is used in the present study, is to screen individuals living in high-risk areas to detect asymptomatic and low parasitaemia infections. The capability of this approach to find asymptomatic *Plasmodium* carriers relies on the sensitivity of the diagnostic tool used [17].

Due to the limited sensitivity of conventional diagnostic tests (microscopy and malaria rapid diagnostic tests: RDTs) [9], and the high cost and technical skills required for polymerase chain reaction (PCR) [18, 19], it is necessary to use molecular methods such as Loop-mediated isothermal Amplification (LAMP). LAMP is a simple and high-sensitive molecular diagnostic technique that is designed for rapid and specific amplification of target DNA at a constant temperature. It can detect 1 to 2 copies of *Plasmodium* DNA per reaction which corresponds to 1 to 2 *Plasmodium* parasites per µl of blood [20]. Unlike PCR, the LAMP method does not require labour-intensive initial DNA extraction, thermocycling during the reaction, multiple turnarounds, high skills, and sophisticated equipment and has a similar sensitivity to PCR [18, 19, 21]. Moreover, the LAMP method is easier, cheaper, and more rapid to perform compared to PCR [19]. In addition, all the LAMP reagents for malaria kits (Loopamp^™^ MALARIA Detection Kit, Eiken Chemical, Co. Ltd., Japan) can be stored at room temperature.

Although new interventions, including targeted drug administration (TDA) have been introduced for malaria control in southern Lao PDR since 2022, no recent community-based study has been conducted to assess the prevalence of asymptomatic malaria infections and the associated factors, which can help to better understand risk profiles. Furthermore, even though several studies recommend using active case detection and the LAMP method to detect asymptomatic *Plasmodium* carriers in countries moving towards disease elimination [4, 9, 15, 22], no report is available on proactive case detection with the LAMP method to find clusters of asymptomatic malaria individuals among risk populations in Lao PDR.

Therefore, this study aimed to assess the prevalence of asymptomatic malaria infections and risk factors associated with it, using the LAMP method, alongside evaluating protective measures and risk behaviour among the rural community of Nong District. The findings of the present study should contribute to a better understanding of asymptomatic malaria infections in a malaria-endemic community of the Lao PDR. Thus, it ultimately contributes to accelerating malaria elimination by 2030 set by the Lao government and the WHO.

## Methods

### Study design

This was a community-based cross-sectional survey that assessed the prevalence of asymptomatic *Plasmodium* infections and factors associated with it, as well as protective measures and risk behavior among the rural community of Nong District.

### Study site and population

This study was carried out during the dry season from the 1st to the 6th of March 2024 in Nong District located in Savannakhet Province.

Savannakhet Province is located ~ 600 km south of Vientiane Capital, Lao PDR. With an area of 21,774 km^2^, Savannakhet is the largest province by population, representing nearly 14% of the country’s population. Nong District is one of the 15 Districts of the Province. Nong District has a high landscape of 280 m above sea level and a 1,824 km^2^ area representing 8.9% of the province’s total surface. The district borders Phine District and Sepon District in Savannakhet Province, Ta Oy District and Sa Mouay District in Salavan Province, and Heuang Hoa District in Vietnam. The topography of the district is mountainous, with dense forests and a temperature range of 15–38 degrees Celsius. With 68 villages and eight ethnic groups, the district has a population of 34,109 people with almost 90% of the workforce practicing agriculture (animal rearing and farming). The district has eight health centres (HC) and one district hospital. Nong District is among the districts with substantial malaria cases as reported by previous studies [4, 8].

According to the Centre of Malariology, Parasitology, and Entomology (CMPE), the Lao Ministry of Health, Lao PDR, in 2022 and 2023, 140 and 30 malaria cases were recorded, respectively, in the district by health centres through passive case detection. The annual parasite incidence (API) in the district was 4.1 cases per 1000 population in 2022, and 0.88 case per 1000 population in 2023, with the prevalent species being *Plasmodium falciparum* and *Plasmodium vivax.*

**Fig. 1:**
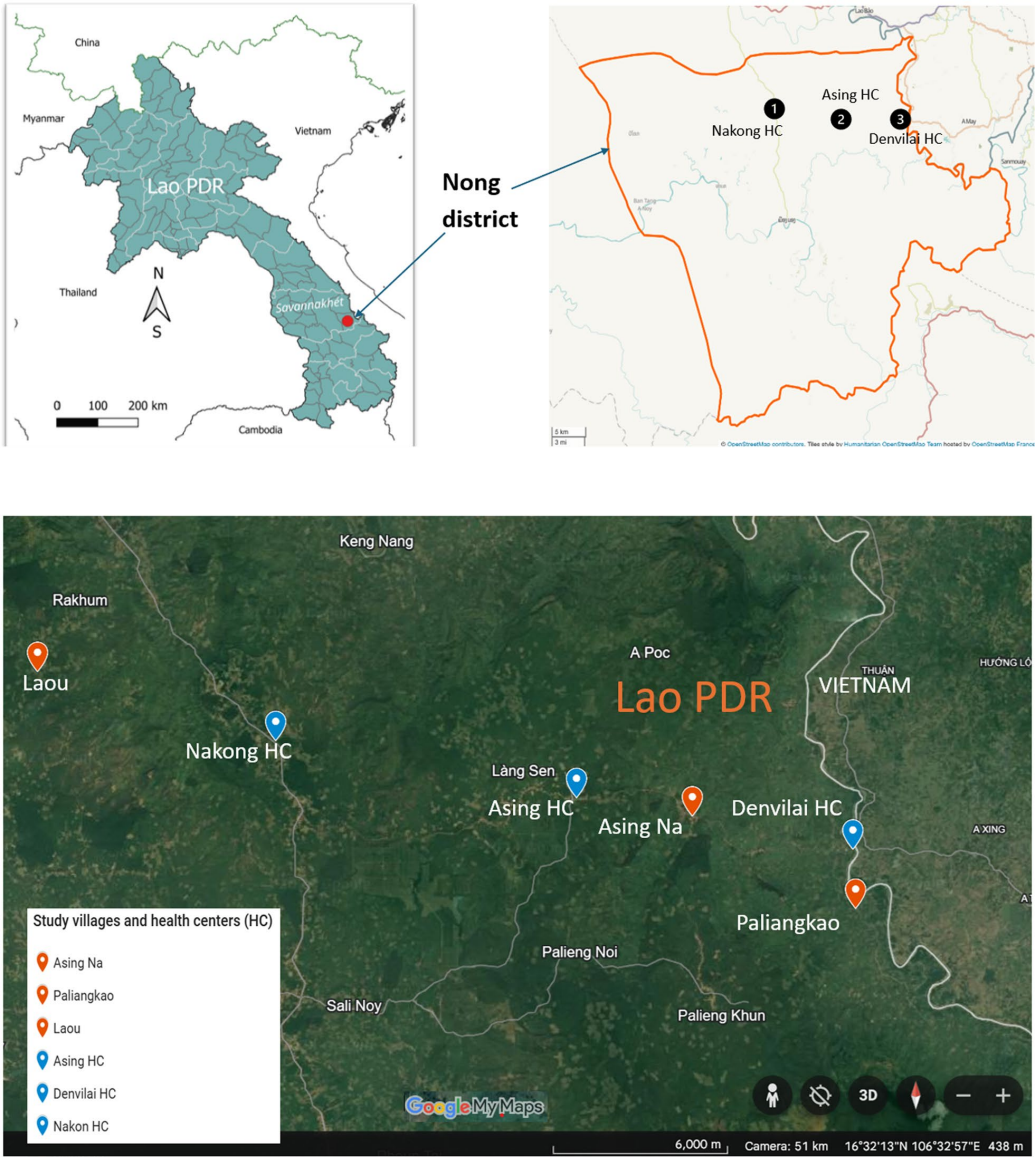
Study site

The survey was conducted in 3 villages (Fig. 1), namely Asing Na, Paliangkao, and Laou, located in the catchment area of three health centres (Asing HC, Denvilai HC, and Nakong HC). These villages were selected on the following basis:

- The predominance of ethnic minorities (Mangkong and Tri) who are known to be vulnerable to malaria,
- And the absence of targeted drug administration (TDA).

The study subjects were participants living in the selected study sites. The inclusion criteria are all residents willing to participate in the research except children under 2 years of age and patients undergoing treatment. A questionnaire on malaria protective measures and risk behavior was administered to participants aged 13 years and above. In rural Lao context, children by the age of 13 engage in various activities including farming [23] and they are likely to respond to the research questionnaire appropriately.

### Sample size calculation

Based on a study conducted with participants living in Nong District in 2017 which revealed 4.8% of asymptomatic infections prevalence at baseline [24] and given the current decrease in malaria cases, we hypothesized that using the molecular method (i.e., LAMP), there would be 2% of asymptomatic malaria infections. Using Easy R (EZR) software (version 1.61, Saitama Medical Centre, Jichi Medical University, Japan) with the following assumed details for sample size calculation, Proportion: 2%; Confidence level: 95%; Confidence interval width: 1.5%, the minimum required sample size was 1339. However, the sample size approved by the National Ethics Committee for Health Research (NECHR), Lao PDR, was 725. This was due to practical constraints, mainly resource limitations and the availability of participants in the targeted villages during data collection. Hence, the confidence interval width was adjusted to 2.0% with the same assumptions of the proportion of asymptomatic *Plasmodium* carriers (2.0%) and the confidence level (95%). As a result, the minimum sample size required was 753.

### Ethical consideration and consent to participate

This study proposal was approved by the National Ethics Committee for Health Research of Lao PDR (approval number: 01/NECHR, 16/1/2024) and the Ethics Committee of the University of the Ryukyus for Medical and Health Research Involving Human Subjects, Japan (Permit number: 23-2250-00-00-00, 20/2/2024). The study was explained to participants present at the survey site using a study information sheet. Written informed consent was obtained from adult participants (≥ 18 years), assent from minors aged 13–17 years, and parental or guardian consent was also obtained for children aged 2–12 years.

### Data collection

The research team consisted of six local health personnel and two staff from Institut Pasteur du Laos who were trained before the survey began, and a team made of three persons from the University of the Ryukyus, Japan. The survey questionnaire was translated from English into Lao language by a native Lao speaker and independently double-checked by another local health personnel. This questionnaire was then structured using Epicollect5 and entered into tablet computers which were used to interview participants.

Before the start of the study, village household lists were prepared with the help of local health centre staff by recording residents’ information in each household in the targeted villages. Prior to the survey, the head of the villages and village health volunteers conducted a sensitization on the study to mobilize participants. Before the survey, a pre-test was conducted with 28 voluntary participants in a non-target village to adjust the questionnaire. During the survey, participants were invited to come in family groups to the house of the village head where the household lists were used for participant registration. The study was explained and written informed consent was obtained from participants willing to join the survey.

Each participant was assigned a unique 5-digit ID. Information on demographics, clinical history, and malaria preventive measures was collected, and tympanic temperature was measured. Village health volunteers helped interpret the survey  questions for the participants who could not speak the Lao language.

Then approximately 250 µl of finger-prick blood samples were collected: 50 µl were used to perform thick and thin blood smears, and 200 µl were air-dried on filter papers (Whatman^™^ FTA^®^ Classic Card, GE Healthcare Life Sciences, UK) for the LAMP tests. Each blood smear and filter paper were identified with the participant’s unique ID. The filter papers with dried blood spots were packed in sealable bags with silica gel and the thick and thin blood smears were packed in labelled slide boxes. All the samples were conveyed to the Nong District Hospital, where they were stored at room temperature, and then to Institut Pasteur du Laos in Vientiane capital the following day.

### Laboratory procedures

#### Light microscopy

The blood films were prepared as a gold standard to quantify parasite density and identify species as well. Blood smear staining was conducted in Nong District Hospital. After fixing the thin smear with methanol, both smears (thin and thick) were stained with a 10% Giemsa working solution. Thick blood smears were examined under light microscopy with immersion oil (100 × magnification) independently by two experienced microscopists (one medical laboratory technician and one research microscopist from Institut Pasteur du Laos). Two hundred high-power fields were examined before negative results were reported. Any discordance such as a positive result from one microscopist and a negative result from another was resolved by a third experienced microscopist.

#### DNA extraction with the procedure for Ultra-Rapid extraction method (LAMP PURE DNA extraction)

Deoxyribonucleic acid (DNA) was extracted from the dried blood samples on filter paper using Loopamp^™^ PURE DNA Extraction Kit (Eiken Chemical Co., Ltd., Japan) according to the manufacturer’s supplied protocol at Institut Pasteur du Laos. Briefly, six millimetres of dried blood spot was punched directly into the heating tube and 30 μl of 334 mM NaCl solution were added. After mixing, the tube was placed into the heating block of the Loopamp LF-160 (75 °C for 5 min). After removing and cooling for 2 min, the heating tube was screwed to the absorbent tube, flicked, and swung 20 times to mix the extraction solution with the adsorbent powder. The porous adsorbent powder removes proteins and other contaminants from the extraction solution that might inhibit nucleic acid amplification. Then, the extracted DNA was transferred to the LAMP reaction tube immediately.

#### DNA amplification with LAMP method

Pan LAMP assay detecting all human-infecting *Plasmodium spp* (Loopamp^™^ Malaria Pan Detection Kit, Eiken Chemical Co., Ltd., Japan) (Malaria Pan (genus)-specific primers: sensitivity 97.0%; specificity 99.2%) was used to amplify *Plasmodium* DNA. The DNA amplification with the LAMP method is performed at a constant temperature (isothermal conditions) using one enzyme and four primers recognizing six distinct regions on the target [25]. The Malaria Pan (genus)-specific primers are designed to detect the mitochondrial DNA with a well-conserved base sequence (18S ribosomal RNA) in the four most widespread malaria-causing *Plasmodium* species (*P. falciparum*, *P. vivax*, *P. ovale,* and *P. malariae*). Therefore, in the presence of parasite DNA, one of the primers binds to the complementary or matching DNA from the parasite.

Before the reaction starts, 30 μl of the extracted DNA were dispensed into the LAMP reaction tube, which has a cap containing the dried form of the strand displacement DNA polymerase, deoxynucleotide triphosphates (dATP, dCTP, dGTP, and dTTP), magnesium chloride, reaction buffers, and Malaria Pan (genus)-specific primers. This dried LAMP reagent [Malaria Pan detection reagent (dMAL Pan)] is dissolved by inverting and shaking the reaction tube. Then, the reaction tube is incubated at 65 °C for 40 min and at 80 °C for 5 min (enzyme inactivation) using Loopamp LF-160 Homeothermal Equipment with ultra-violet (UV) lamp (Loopamp LF-160—Eiken Chemical Co. Ltd., Japan). The DNA is amplified by the strand displacement DNA polymerase per the LAMP reaction. Negative and positive controls were included in each run.

#### Visual detection of LAMP result, specific species, and test validity

The detection of amplified DNA was done visually under UV light. A reaction is positive when there is a fluorescent green light in the reaction tube, and negative when there is not [25]. If any of the controls (positive and negative) were not valid, all the reactions were invalid and should be repeated. Positive samples were retested using the *Plasmodium falciparum* detection kit (Loopamp^™^ Malaria *Pf* Detection Kit) and *P. vivax* detection kit (Loopamp^™^ Malaria *Pv* Detection Kit) for species identification.

### Operational definition of asymptomatic malaria

Asymptomatic *Plasmodium* infection was defined in this study as the detection of malaria parasite DNA in non-febrile participants with tympanic temperature < 37.5 °C, with no malaria-related symptoms such as headache, chills, joint pains, vomiting, etc., 2 weeks before and at the time of the survey [26].

### Data management and analysis

Interview data were extracted with a spreadsheet from the Epicollect 5 data hosting server. Data cleaning was done by removing duplicates, checking inconsistencies, and standardizing variable names and formats. Among 628 participants registered, 4 non-interviewed participants and 2 interviewees were excluded from data analysis for not providing blood samples.

The explanatory variables were gender, age grouping, temperature, occupation, having experienced malaria symptoms 2 weeks ago, presence of malaria symptoms during the survey, being in the forest in the past month, night work in the forest, and use of protective measures; the outcome variable was malaria infection status determined by the LAMP method. The statistical analysis was performed using EZR software. The continuous variables (age and temperature) were summarized into the median (interquartile range). Mann–Whitney *U* test was performed to compare the medians of different age groups and temperature groups. For categorical variables, Fisher’s exact test was performed to determine the statistical association between the explanatory variables and the outcome variable (malaria infection status determined by LAMP method). A *p* value of < 0.05 was accepted as a statistically significant association.

## Results

### Characteristics of study participants

In the present study, 622 participants were enrolled. These participants came from 164 households. Among the three villages, Asing Na had the highest number of participants, 44.0% (274/622), followed by Laou, 40.2% (250/622), and Paliangkao, 15.8% (98/622) (Table 1). Females represented the majority (58.4%) of the total participants. The study population age ranged from 2 to 89 years with a median age of 16 years (Inter-quartile range: IQR = 9 to 33). The predominant participants were in the age group of 15 years and above, representing 53.8% of all the participants. Nine participants had a fever at the time of the survey (body temperature > 37.5ºC); four participants’ body temperatures were missed.

**Table 1:** Characteristics of study participants

Characteristics	Total samples		LAMP positive		LAMP negative		*P*-value
	*n* = 622	%	*n* = 13	%	*n* = 609	%	
Study sites							
Asing Na	274	44.0	4	30.8	270	44.3	0.08
Paliangkao	98	15.8	0	0.0	98	16.1	
Laou	250	40.2	9	69.2	241	39.6	
Gender							
Male	258	41.5	7	53.8	251	41.2	0.4
Female	363	58.4	6	46.2	357	58.6	
Not determined	1	0.1	0	0.0	1	0.2	
Age							
Median (Interquartile range)	16 (9–33)		43 (16–48)		16 (9–33)		0.02
Age group							
< 5 years	42	6.8	0	0.0	42	6.9	0.1
5–14 years	244	39.2	2	15.4	242	39.7	
≥ 15 years	335	53.8	11	84.6	324	53.2	
Not determined	1	0.2	0	0.0	1	0.2	
Tympanic temperature							
Median (Interquartile range)	36.5 (36–36.9)		36.6 (36.4–36.8)		36.5 (36–36.9)		0.64
< 37.5ºC	609	98.0	12	92.3	597	98.0	> 0.99
≥ 37.5ºC	9	1.6	0	0.0	9	1.5	
Not determined	4	0.4	1	7.7	3	0.5	

**Fig. 2:**
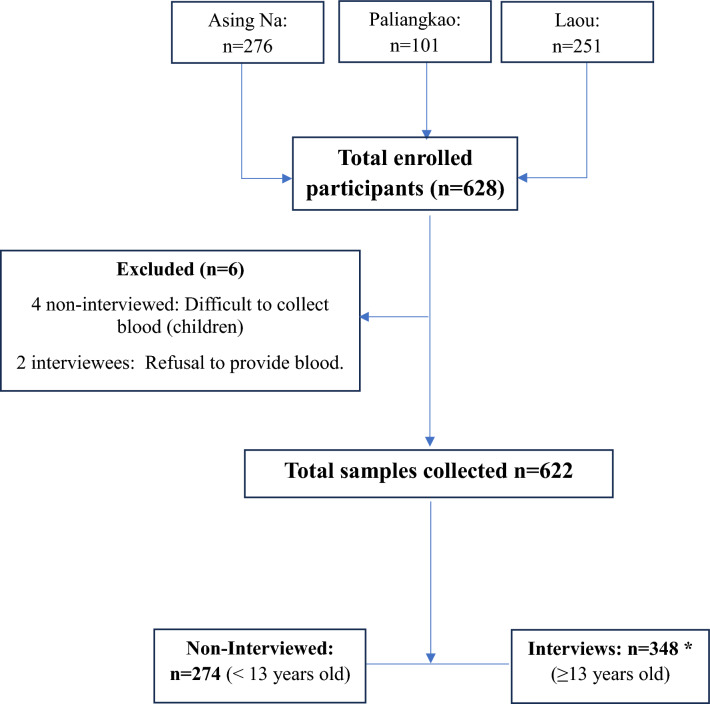
Study flow chart, (*): 350 interviews were conducted; 2 were excluded for refusing to provide blood samples

Out of the total participants, 350 interviews were conducted (two excluded from the analysis as they did not provide blood samples) with participants aged 13 years and above (Fig. 2). Most of the interviewees had no formal education (69.2%) and were farmers (96.6%) (Table 2).

**Table 2:** Characteristics of interviewed participants (*n* = 348)

Characteristics	Total samples		LAMP positive		LAMP negative		*P*-value
	*n* = 348	%	*n* = 11	%	*n* = 337	%	
Gender							
Male	140	40.2	6	54.5	134	39.8	0.4
Female	208	59.8	5	45.5	203	60.2	
Age							
Median (Interquartile range)	29.5 (19.8 to 46)		43 (28.5 to 48)		29 (19 to 46)		0.2
Age group							
13–14	29	8.3	0	0	29	8.6	0.6
≥ 15	319	91.7	11	100	308	91.4	
Educational attainment							
No formal education	241	69.2	9	81.8	232	68.8	0.9
Primary school	79	22.7	2	18.2	77	22.8	
Secondary school and above	28	8.1	0	0.0	28	8.3	
Occupation							
Farmers	336	96.6	11	100	325	96.4	> 0.99
Others (Trader, student, teacher, no occupation)	12	3.4	0	0.0	12	3.6	
Clinical history							
Symptoms 2 weeks before blood collection							
Had	164	47.1	4	36.4	160	47.5	0.6
Did not have	184	52.9	7	63.6	177	52.5	
Symptoms on the day of blood collection							
Had	55	15.8	1	9.1	54	16	> 0.99
Did not have	293	84.2	10	90.9	283	84	
Bed net use in the village							
Yes	313	90.0	9	81.8	304	90.2	0.3
No	35	10.0	2	18.2	33	9.8	
Frequency of bed net use(*n* = 313)							
Always	277	88.5	7	77.8	270	88.8	0.3
Sometimes	36	11.5	2	22.2	34	11.2	
Have been to the forest in the past month							
Yes	263	75.6	8	72.7	255	75.7	0.7
No	85	24.4	3	27.3	82	24.3	
Night work in the forest (for those who have been to the forest *n* = 263)							
Yes	56	21.3	1	12.5	55	21.6	> 0.99
No	204	77.6	7	87.5	197	77.2	
Not determined	3	1.1	0	0.0	3	1.2	
Bed net use in forest/rice field							
Yes	48	13.8	1	9.1	47	13.9	0.5
No	82	23.6	1	9.1	81	24.0	
Don’t sleep in the forest	218	62.6	9	81.8	209	62.0	
Other protective measures in the forest or rice field apart from bed net							
Mosquito repellent	21	6.0	0	0.0	21	6.2	0.4
No measure	208	59.7	6	54.5	202	59.9	
Long clothes	112	32.2	4	36.4	108	32.0	
Other (make fire, etc.)	7	2.0	1	9.1	6	1.8	
Travel to another district in the past month							
Yes	38	10.9	0	0.0	38	11.3	0.6
No	310	89.1	11	100.0	299	88.7	

### Malaria infections detected by microscopy

The observation of thick blood smears under light microscopy resulted in no malaria-positive case in the present study. The observation of thin blood smears of the LAMP positive samples under light microscopy also resulted in no malaria-positive case.

### Prevalence of asymptomatic *Plasmodium* infections

The performance of the LAMP assay on the total 622 blood samples yielded 13 *Plasmodium spp.* infections clustered primarily among adults (median age: 43, interquartile range: 16–48; *p* < 0.05) (Table 1). *Plasmodium vivax* accounted for 15.4% (2/13). The remaining species could not be identified*.* Overall, the prevalence of asymptomatic *Plasmodium* infections was 1.8% (11/622; 95% CI [0.9–3%]). Of the total 13 malaria cases, Laou village had the highest prevalence of infections, 3.6% (9/250) followed by Asing Na, 1.5% (4/274). No malaria infection was detected among the participants from Paliangkao village (0/98) (Table 1). Two school-age children (6 and 8 years) tested malaria-positive with no fever; males represented 53.8% of malaria infections; The body temperature of one malaria-positive case (1/13, adult, male) was missed and therefore was classified as neither symptomatic nor asymptomatic; 92.3% (12/13) of the positive cases had no fever (tympanic temperature < 37.5 °C) at the time of the survey (Table 1). Only one participant (adult) had symptoms (headache and chills) 2 weeks before the survey, and headache on the day of the survey, and tested positive for malaria with no fever. Hence, he was excluded from asymptomatic malaria.

### Trend of asymptomatic *Plasmodium* infections

In this dataset, there was no statistically significant association between the explanatory variables and asymptomatic infections. However, 81.8% (9/11) of the asymptomatic infections were found among farmers aged 15 years and above (Table 3). In addition, among malaria-positive cases, no cohabitant or household member was revealed to have malaria infection.

**Table 3:** Summary of the prevalence of asymptomatic malaria

Characteristics	Total samples	Total positive infections (2.1%, 13/622)				
		Symptomatic and undetermined status		Asymptomatic (1.8%, 11/622)		*P*-value
	*n* = 622	*n* = 2	%	*n* = 11	%	
Gender						
Male	258	2	100	5	45.5	0.46
Female	363	0	0.0	6	54.5	
Not determined	1	0	0.0	0	0.0	
Age						
Median (Interquartile range)	16 (9–33)	40 (38.5–40.5)		43 (15.5–48)		> 0.99
Age group						
< 5 years	42	0	0.0	0	0.0	> 0.99
5 to 14 years	244	0	0.0	2	18.2	
≥ 15 years	335	2	100	9	81.8	
Not determined	1	0	0.0	0	0.0	
Tympanic temperature (34ºC–38.6°C)						
Median (Interquartile range)	36.5 (36–36.9)	36.8 (36.8)		36.5 (36.4–36.8)		0.6
< 37.5ºC	609	1	50.0	11	100	0.15
≥ 37.5º	9	0	0.0	0	0	
Not determined	4	1	50.0	–	–	
Occupation						
Farmers	336	2	100	9	81.8	> 0.99
Others	286	0	0.0	2	18.2	
Symptoms 2 weeks before the survey and on the day of the survey						
Yes	44	1	50.0	0	0.0	0.15
No	304	1	50.0	11	100	

### Protective measures and risk behaviour

Ninety percent of the participants (313/348) used mosquito bed nets when sleeping in the village, and 88.5%  of them reported using it always (Table 2). A month before the survey, 75.6% of the participants had been to the forest and they represented 72.7% (8/11) of malaria cases; 21.3% of them worked at night in the forest. Among forest goers, 23.6% reported not using mosquito bed nets in the forest. In the present study, participants who did not use bed nets provided the following reasons: “not having a bed net at all”, “no extra bed net for the forest”, “hot inside the net”, “difficulty in carrying the bed net to the forest”, and “the net itches the body”. Few participants (38) among interviewees travelled to other districts such as Xepon, Xiengkhuang, and Champhonne Districts as well as the neighbouring country Vietnam.

## Discussion

### Prevalence of asymptomatic *Plasmodium* infections

This first community-based proactive case detection using the malaria LAMP technique assessed the prevalence of asymptomatic *Plasmodium* infections in three remote villages of Lao PDR.

While a significant decrease in malaria cases has been reported in the Nong District in the past 2 years (2022 and 2023), the main finding of this study is the 1.8% prevalence of asymptomatic *Plasmodium* infections. This shows that asymptomatic *Plasmodium* parasite carriers are still considerable in the two of the study villages (Asing Na and Laou). The prevalence of asymptomatic infections in this study corroborates previous studies conducted in similar malaria-endemic settings with low transmission in the northwestern border of Thailand where 2.3% of submicroscopic infections were reported [27] using LAMP, and in eastern Myanmar where 2.3% of asymptomatic *Plasmodium* infections were reported [28].

Although the TDA is conducted by the Lao government in some villages in Nong District, it is not implemented in the three villages studied. In addition, only two malaria cases were recorded in Laou in 2022, and no malaria case was recorded by local healthcare facilities or village health volunteers from 2023 until March 2024 (time of this survey). However, Laou is the village where most of the infections (3.6%, 9/250) were found in this study. This shows how healthcare facilities adopting passive case detection miss malaria infections which could lead to the disease spread. This is in line with the situation in Khammouane province, where a malaria outbreak (116 cases) occurred in Nakay District in June-July 2023, a place where only one malaria case was reported from January 2022 until June 2023 [CMPE, the Ministry of Health, Lao PDR].

These results indicate that *Plasmodium* parasites are circulating within certain areas and specific populations that constitute the parasite's reservoir and could contribute to its spread. As these individuals are not aware of their *Plasmodium* infection status due to the absence of symptoms, they do not seek treatment from healthcare facilities or village health volunteers [29]. This is consistent with previous studies in the Greater Mekong Subregion [27, 30]. Therefore, targeting those individuals at risk for screening using the LAMP method and treatment every 6 months or year (depending on resource availability) would be an essential approach for maintaining zero malaria cases to prevent the potential spread of the disease and achieve its elimination.

### Heterogeneity of *Plasmodium* infections

Substantial differences in *Plasmodium* infections have been observed among the three villages studied. Although Asing Na is the village with more participants in this study, 44.1% (274/622), only 1.5% (4/274) of malaria cases were detected. On the other hand, Laou is the village with most of the malaria infections [3.6% (9/250)], while no *Plasmodium* parasites were detected among participants in Paliangkao village. These differences could be explained by environmental factors such as the proximity of villages to the forest and human exposure to *Anopheles* mosquitoes due to occupations (working in the forest). This could substantiate the high proportion of asymptomatic infections in Laou village, since 48% of the participants had been to the forest a month before this study compared to 39.5% and 12.5% in Asing Na and Paliangkao respectively. This heterogeneity is in agreement with previous studies in Savannakhet [12], in northwestern Thailand [27], and in Western Cambodia [31].

### Age, occupation, and trend of asymptomatic *Plasmodium* infections

The clustering of more than 80% of asymptomatic *Plasmodium* infections amidst young and adult farmers (≥ 15 years) could be explained by the fact that older adolescents and people in the middle age group are likely to be infected with malaria due to their occupation, such as working in the forest or rice field. Thus, they could have developed immunity to malaria following frequent exposure to *Plasmodium* parasites, which makes them prone to asymptomatic *Plasmodium* infection. This trend is consistent with the study on the northwestern border of Thailand [27], which reported a peak risk of asymptomatic *Plasmodium* infections to be between 31 and 45 years. The study in eastern Myanmar [28] also reported asymptomatic *Plasmodium* infections to be limited to people older than 17 years.

### Absence of asymptomatic *Plasmodium* infections among household members or cohabitants

Malaria infections were detected in 13 different households. However, no household member or cohabitant of an infected individual was found to be malaria-positive. This may be explained by the fact that malaria transmission generally occurs outside households [27] in the Greater Mekong Subregion [32, 33]. This suggests that younger children would be less exposed to malaria vectors if they did not visit forests or farms. In addition, 90% of the participants reported using mosquito bed nets when staying in the villages. Although statistically, there was no significant association between occupation and asymptomatic infections in this study, all the asymptomatic infections were found among farmers aged 15 years and older, except two school-aged children (6 and 8 years). Moreover, 75.6% of interviewed participants have been to the forest a month before the survey which also may expose them to malaria infection. The fact that the survey was conducted during the dry season when the number of *Anopheles* mosquitoes in the village was low, could be another reason why no intra-household transmission was observed.

In addition, the lack of protective measures usage among at-risk populations is considerable, making it a concern in the context of near malaria elimination. To ensure the break of the transmission chain despite being low, reinforcing   the use of malaria protective measures is essential.

### Unidentified *Plasmodium* species

Out of 13 positive *Plasmodium spp*. infections, only two cases were *Plasmodium vivax.* The remaining species could not be identified*.* This could be explained by the fact that although the Pan LAMP assay is highly sensitive, it can detect only the DNA of the *Plasmodium* genus without specifying the species and, therefore, requires species-specific assays for further identification. Hence, in this study, we used two species-specific LAMP kits (*P. falciparum* detection kit and *P. vivax* detection kit) for species identification of positive samples, as in the study area, only these two *Plasmodium* species are prevalent. The non-identification of *P. falciparum* or more cases of *P. vivax* could be due to possible low parasite density in the finger prick capillary blood samples, which could make the target DNA of a particular species insufficient for species-specific assay to amplify. This non-identification of *Plasmodium* species has also been reported in other studies that have used other molecular methods such as PCR [12, 34]. In addition, the non-identified species could also possibly be other *Plasmodium* species than *P. falciparum* and *P. vivax.* Further studies would be necessary to explore this possibility.

### Limitations

The present study has some limitations. The small sample size might affect the precision of the estimates. In addition, the study was conducted during the dry season, which is a period when malaria transmission is low in Lao PDR. Another limitation is the fact that interviews were conducted with participants aged 13 years and above and the study was limited to three villages in the district. This low geographical coverage could affect the generalizability of the study. Furthermore, the multivariate analysis was not conducted due to the low proportion of the asymptomatic infections, making the analysis unsuitable. The little representation of infections in the outcome variables may lead to insufficient statistical power [35].

## Conclusion and recommendation

The current study revealed 1.8% (11/622) prevalence of asymptomatic *Plasmodium* infections in rural endemic areas of Lao PDR. The majority of the asymptomatic infections were clustered among older adolescents and adult farmers with an important proportion of these at-risk populations not using protective measures.

These findings showed that malaria reservoirs are notable with a lack of use of protective measures, which could threaten malaria control and elimination efforts. Therefore, malaria elimination in Lao PDR by 2030 would need interventions targeting high-risk adult populations for screening with sensitive tools coupled with sensitization on protective measures and asymptomatic *Plasmodium* infection.

## Supplementary Information

Supplementary material 1.

## Data Availability

The data generated and analysed during the current study are available from the corresponding author upon reasonable request.

## Abbreviations

DNA: Deoxy-ribonucleic Acid
FTA: Flinders Technology Associates
HC: Health centre
ID: Identifier
INTs: Insecticide-treated Nets
LAMP: Loop-mediated Isothermal Amplification
Lao PDR: Lao People’s Democratic Republic
NECHR: National Ethics Committee for Health Research
P. f: Plasmodium falciparum
P.v: Plasmodium vivax
PACD: Proactive case detection
RACD: Reactive case detection
RDTs: Rapid diagnostic tests
RNA: Ribonucleic acid
TDA: Targeted drug administration
UV: Ultra-violet
WHO: World Health Organization
