# Supplementary material for: Asymptomatic malaria infections and associated risk factors in malaria-eliminating settings of Nong District, Savannakhet Province, Lao People’s Democratic Republic
Source: Trop Med Health. 2025 Feb 19;53:24. doi: 10.1186/s41182-025-00702-y (PMC11841228; doi:10.1186/s41182-025-00702-y)
Supplement: Supplementary file 1 — Supplementary material 1. [file 41182_2025_702_MOESM1_ESM.docx]

**Supplementary information**

Table 4: Asymptomatic infection by study site

|  | Total samples | Symptomatic and undetermined infections | | Asymptomatic infections | | P-value |
| --- | --- | --- | --- | --- | --- | --- |
| **Study site** | n= 622 | n = 2 | % | n = 11 | % |  |
| Asing Na | 274 | 0 | 0.0 | 4 | 36.4 |  |
| Paliangkao | 98 | 0 | 0.0 | 0 | 0.0 | > 0.99 |
| Laou | 250 | 2 | 100 | 7 | 63.6 |  |

## Questionnaire for the participants

| **Interview on sociodemographic** | | |
| --- | --- | --- |
|  | Participant’s ID: | |
|  | Gender: □^1^  Male □^2^ Female | |
|  | Age: | |
|  | Educational attainment | □^1^ No formal education  □^2^ Primary school □^3^ Secondary school and above  □^3^ Other (Specify) |
|  | Occupation | □^1^ Farmer □^2^ trader □^3^  Forestry  □^4^ Student □^5^ Housemaker  □^6^ No occupation (elderly or kids)  □^7^ teacher □^8^ police □^9^ Soldier  □ Other (specify) |

| **Interview on Clinical history** | | |
| --- | --- | --- |
|  | Did you have a fever in the past 2 weeks? | □^1^ Yes □^2^ No |
|  | Did you have a headache in the past 2 weeks? | □^1^ Yes □^2^ No |
|  | Did you have chills in the past 2 weeks? | □^1^ Yes □^2^ No |
|  | Did you have vomiting in the past 2 weeks? | □^1^ Yes □^2^ No |
|  | Did you have joint pain in the past 2 weeks? | □^1^ Yes □^2^ No |
|  | Do you have any symptoms today? | □^1^ Yes  □^2^ No (if answered skip Q13) |
|  | What symptoms did you have today? | □^1^ Fever □^2^ Headache □^3^ Chills  □^4^ Joints pain □^5^ Vomiting  □ Other (specify: □^6^ Stomach ache □^7^ Sore throat □^8^ Running nose  □^9^ Cough, □^10^Fatigue |
|  | Do you use mosquito bed nets when you sleep in the village? | □^1^ Yes (if answered skip Q16)  □^2^ No (if answered skip Q15) |
|  | How often do you use mosquito bed nets when you sleep in the village? | □^1^ Always □^2^ Sometimes |
|  | Why don’t you use mosquito bed nets? | □^1^ I don’t have  □^2^ It is hot inside  □^3^ The net burns my body  □^4^ Other (specify: ) |
|  | Do you use mosquito bed nets when you sleep in the rice field or forest? | □^1^ Yes (if answered skip Q19)  □^2^ No (if answered skip Q18)  □^3^ I don’t sleep in the rice field or forest (if answered skip Q18 & 19) |
|  | How often do you use mosquito bed nets when you sleep in the rice field or forest? | □^1^ Always □^2^ Sometimes |
|  | Why don’t you use mosquito bed nets when you sleep in the forest? | □^1^ I don’t have  □^2^ I don’t have extra nets for rice fields or forest  □^3^ Difficult to carry nets to the rice field or forest  □^4^ It is hot inside  □^5^ The net burns my body  □ Other (specify: □^6^ Forget to bring the net to the forest. □^7^ Don’t want to use bed net |
|  | Do you use mosquito repellent? | □^1^ yes □^2^ No |
|  | Have you been to the forest in the past 1 month? | □^1^ Yes  □^2^ No (if answered skip Q22) |
|  | Do you work in the rice field or forest at night? | □^1^ Yes □^2^ No |
|  | What other measures do you use to protect yourself in the forest or rice field apart from bed nets? | □^1^ I use mosquito repellent  □^2^ No protective measure  □^3^ I wear clothes that cover all my body  □^4^ Other (specify: □^5^ Make fire □^6^ Cover all the body with blanket |
|  | Have you travelled to another district in the past 1 month? | □^1^ Yes  □^2^ No (if answered skip Q25) |
|  | Which district did you travel to? | □^1^  □^2^  □^3^  □^4^ |
